# Mobilization of lymphatic endothelial precursor cells and lymphatic neovascularization in primary Sjögren's syndrome

**DOI:** 10.1111/jcmm.12793

**Published:** 2016-02-01

**Authors:** Alessia Alunno, Lidia Ibba‐Manneschi, Onelia Bistoni, Irene Rosa, Sara Caterbi, Roberto Gerli, Mirko Manetti

**Affiliations:** ^1^ Rheumatology Unit Department of Medicine University of Perugia Perugia Italy; ^2^ Department of Experimental and Clinical Medicine Section of Anatomy and Histology University of Florence Florence Italy

**Keywords:** primary Sjögren's syndrome, minor salivary glands, lymphatic endothelial precursor cells, lymphangiogenesis, lymphvasculogenesis, Th17 cells, IL‐17

## Abstract

Although lymphatic neovascularization may be a key feature of chronic inflammation, it is almost unexplored in primary Sjögren's syndrome (pSS). A recent study revealed a pro‐lymphangiogenic function of interleukin (IL)‐17, a leading player in pSS pathogenesis. The aims of the study were to investigate lymphangiogenic mediators and lymphatic vasculature in pSS, as well as their possible association with IL‐17. Circulating lymphatic endothelial precursor cells (LEPCs) and Th17 cells were enumerated in pSS patients and healthy donors. VEGF‐C and IL‐17 levels were assessed in paired serum samples. Lymphatic vasculature, VEGF‐C/VEGF receptor (VEGFR)‐3 and IL‐17 were evaluated in pSS minor salivary glands (MSGs) and compared with normal and non‐specific chronic sialadenitis (NSCS) MSGs. Circulating LEPCs were expanded in pSS and correlated with circulating Th17 cells, IL‐17 and VEGF‐C. In pSS MSGs, a newly formed lymphatic capillary network was found within periductal inflammatory infiltrates and the number of interlobular lymphatic vessels was significantly increased compared with normal and NSCS MSGs. Strong VEGF‐C expression was detected in pSS ductal epithelial cells and periductal inflammatory cells. Numerous VEGFR‐3^+^ infiltrating mononuclear cells were exclusively observed in pSS MSGs. VEGFR‐3 expression was strongly increased in lymphatic capillaries of pSS MSGs. IL‐17^+^ inflammatory cells were preferentially observed around lymphatic vessels in pSS MSGs. This study supports the notion that lymphvasculogenesis and lymphangiogenesis are active in pSS, thereby unmasking a novel aspect of disease pathogenesis. In addition, our results suggest another possible pathogenic role of IL‐17 in pSS, further supporting its therapeutic targeting in this disease.

## Introduction

Primary Sjögren's syndrome (pSS) is a systemic autoimmune disorder characterized by chronic inflammation of exocrine glands leading to impaired secretory function 1. Minor salivary gland (MSG) biopsies of patients with pSS, routinely evaluated for diagnostic purposes, have to display, according to commonly used classification criteria 2, cell aggregates with >50 periductal/perivascular mononuclear cells (*i.e*. foci), a pattern defined as focal lymphocytic sialadenitis (FLS). In a subgroup of patients, such inflammatory infiltrates organize in highly specialized ectopic lymphoid structures that resemble germinal centres characteristic of secondary lymphoid organs.

Since neovascularization is a crucial event during inflammation, it has been put forward the hypothesis that it may be also a pathogenic hallmark of pSS 3. Expansion of the blood vessel microvascular bed within inflamed tissues aims at fuelling inflammatory response *via* recruitment of pro‐inflammatory cells and recirculation of pro‐inflammatory soluble mediators. Similarly, lymphatic neovascularization is a key feature of acute and chronic inflammation. However, whether its role is either protective or deleterious is still a matter of debate. Although an increased blood vessel density that parallels the extent of glandular inflammation and an activation of VEGF‐A/VEGF receptor (VEGFR)‐2 and neuropilin‐1 co‐receptor pro‐angiogenic system have been recently described in pSS 4, 5, 6, the lymphatic vessel counterpart is almost unexplored. Only two studies attempted to quantify the lymphatic vascular bed extension in pSS MSGs through the detection of mature lymphatic endothelial cells (ECs), but they yielded conflicting results reporting either no difference or an increase in lymphatic capillaries in patients compared with controls (the latter defined as MSGs without foci) 7, 8. During adulthood, lymphatic vessels originate not only from lymphangiogenesis, namely from pre‐existing lymphatic vasculature, as previously thought, but also from lymphvasculogenesis, the generation of novel vessels *via* precursor cells. The latter event has been recently confirmed and points out the importance of bone marrow–derived lymphatic endothelial precursor cells (LEPCs) in postnatal lymphvasculogenesis 9. Similarly to their blood vascular counterpart (*i.e*. EPCs), LEPCs express CD34 and CD133 on their surface, but while EPCs display VEGFR‐2, and LEPCs express VEGFR‐3 (also known as Flt4) 10. LEPCs isolated from healthy adult peripheral blood are able to proliferate *in vitro* and give origin to mature ECs expressing lymphatic markers such as LYVE‐1 and podoplanin 10. Lymphatic neovascularization *via* both lymphangiogenesis and lymphvasculogenesis is mainly orchestrated by the VEGF‐C/VEGFR‐3 pathway. Interestingly, VEGFR‐3 silencing in LEPCs seems to reduce their proliferation, thereby preventing their differentiation into mature lymphatic ECs *in vitro* 11. During inflammatory processes, VEGF‐C is abundantly released by immune cells, for example, macrophages, leading to mobilization of LEPCs from bone marrow. An expansion of circulating LEPCs has been previously described in spondyloarthritis and spondyloarthritis associated with Crohn's disease 12, but no data are currently available in pSS.

Growing evidence suggests that interleukin (IL)‐17 and, therefore, IL‐17‐producing T cells are leading players in pSS pathogenesis 13, 14. Interleukin‐17 participates in both induction and perpetuation of glandular inflammation and, likely, also in ectopic lymphoid neogenesis 15, 16. A recent study, employing a mouse cornea micropocket model, demonstrated that IL‐17 is able to induce lymphatic neovascularization *via* the VEGFR‐3 pathway fostering LEPC proliferation 17. It is, therefore, conceivable that IL‐17 may display such pro‐lymphangiogenic function also in pSS.

On these premises, the aims of the present study were (*i*) to investigate circulating LEPCs and their possible association with IL‐17 in pSS and (*ii*) to characterize the lymphatic vasculature and the expression of lymphangiogenic mediators in MSGs from patients with pSS compared with non‐specific chronic sialadenitis (NSCS) and normal MSGs.

## Materials and methods

### Patients and healthy donors

Fifteen female patients with pSS classified according to the American‐European criteria 2 and 15 age‐matched healthy female donors (HD) were enrolled for analyses on peripheral blood samples. Clinical and serological records were collected at the time of enrolment. Disease activity was measured using the EULAR Sjögren's syndrome disease activity index (ESSDAI) 18. All patients were receiving topical medications for sicca symptoms and hydroxychloroquine 200 mg/day. None of the patients was taking corticosteroids or immunosuppressive therapies. The study was approved by the local ethics committee, and written informed consent was obtained from each participant in accordance with the declaration of Helsinki.

### Cell isolation and flow cytometry

Peripheral blood mononuclear cells were isolated from pSS patients and HD by gradient separation. For surface staining, fluorescein isothiocyanate (FITC)‐, phycoerythrin (PE)‐ or allophycocyanin (APC)‐labelled anti‐human CD3, CD4, CD8, CD34, CD133, VEGFR‐3 and respective isotypes were used [Becton Dickinson (BD) Biosciences, San Jose, CA, USA]. LEPCs were defined as CD133^+^ VEGFR‐3^+^ cells among CD34^+^ cells within the lymphocyte gate. For the assessment of intracellular IL‐17, cells were stimulated for 6 hrs at 37°C with 25 ng/ml phorbol 12‐myristate 13‐acetate (PMA), 1 mg/ml ionomycin and 0.1 mg/ml brefeldin in complete medium, then surface staining was performed and cells were fixed with 4% paraformaldehyde. Following fixation, cells were permeabilized with 0.1% saponin blocking buffer and stained with Alexa Fluor 647‐labelled anti‐human IL‐17 or respective isotype (BD Biosciences) as described elsewhere 14. Samples were analysed using FACScalibur flow cytometer (BD Biosciences) and CellQuestPro software (BD Biosciences).

### ELISA on serum samples

The concentration of VEGF‐C and IL‐17 was assessed in paired serum samples from the 15 pSS patients. Ten HD served as controls. Commercially available ELISA kits (Quantikine; R&D Systems, Minneapolis, MN, USA) were used according to the manufacturer's instruction.

### Salivary gland specimens

Twenty‐eight female patients with sicca syndrome symptoms underwent labial MSG biopsy for diagnostic purposes, and 5/6 lobules were blindly analysed for each sample. Minor salivary gland samples were fixed in formalin, dehydrated in graded alcohol series and embedded in paraffin. For routine histopathological analysis, MSG sections (3 μm thick) were deparaffinized, rehydrated and stained with hematoxylin and eosin. The presence of FLS, *i.e*. at least one inflammatory focus within 4 mm^2^ of MSG tissue, allowed the diagnosis of pSS 2, 19 in 12 patients. Of the remaining 16 patients, eight displayed normal MSGs and eight displayed a certain degree of MSG inflammation, *i.e*. NSCS, but no evidence of FLS. None of these 16 patients had serological features of pSS; hence, pSS diagnosis was ruled out according to the American‐European criteria 2. Focus score and Tarpley biopsy score (0–4 scale) 20 were performed on hematoxylin‐ and eosin‐stained sections. Cellular infiltrate and lymphoid organization were assessed by immunofluorescence staining of serial MSG sections with antibodies against human CD3 (T‐cell marker), CD20 (B‐cell marker) and CD21 (marker of follicular dendritic cells characteristic of germinal centre‐like structures) as described elsewhere 21.

### Immunofluorescence staining

Minor salivary gland tissue sections (3 μm thick) were deparaffinized, rehydrated and boiled for 10 min. in sodium citrate buffer (10 mM, pH 6.0). Sections were washed in PBS, incubated in 2 mg/ml glycine for 10 min. to quench autofluorescence caused by free aldehydes and then blocked for 1 hr at room temperature with 1% bovine serum albumin (BSA) in PBS. The sections were then incubated overnight at 4°C with the following primary antibodies diluted in PBS with 1% BSA: mouse monoclonal anti‐human podoplanin (D2‐40) (1:50 dilution; catalogue number M3619; Dako, Glostrup, Denmark), rabbit polyclonal anti‐human CD31/platelet‐endothelial cell adhesion molecule‐1 (1:50 dilution; catalogue number ab28364; Abcam, Cambridge, UK), rabbit polyclonal anti‐human VEGF‐C (1:100 dilution; catalogue number bs‐1586R; BIOSS Antibodies, Woburn, MA, USA), rabbit polyclonal anti‐human VEGFR‐3/Flt4 (1:50 dilution; catalogue number AB1875; Chemicon International, Temecula, CA, USA) and rabbit polyclonal anti‐human IL‐17A (1:100 dilution; catalogue number ab136668; Abcam). The day after, the slides were washed three times in PBS and incubated for 45 min. at room temperature in the dark with Alexa Fluor‐488‐conjugated goat antimouse IgG or Rhodamine Red‐X‐conjugated goat anti‐rabbit IgG (Invitrogen, San Diego, CA, USA) diluted 1:200 in PBS with 1% BSA, as secondary antibodies. Double immunofluorescence staining was performed by mixing mouse and rabbit primary antibodies and subsequently mixing fluorochrome‐conjugated secondary antibodies. Irrelevant isotype‐matched and concentration‐matched mouse and rabbit IgG (Sigma‐Aldrich, St. Louis, MO, USA) were used as negative controls. Cross‐reactivity of secondary antibodies was tested in control experiments in which primary antibodies were omitted. Nuclei were counterstained with 4′,6‐diamidino‐2‐phenylindole (DAPI; Chemicon International). Minor salivary gland sections were then mounted with an antifade aqueous mounting medium (Biomeda Gel Mount; Electron Microscopy Sciences, Foster City, CA, USA) and examined with a Leica DM4000 B microscope equipped with fully automated fluorescence axes (Leica Microsystems, Mannheim, Germany). Fluorescence images were captured with a Leica DFC310 FX 1.4‐megapixel digital colour camera equipped with the Leica software application suite LAS V3.8 (Leica Microsystems). All images were captured using the same exposure time. Densitometric analysis of the intensity of immunofluorescent staining was performed on digitized images using ImageJ software (NIH, Bethesda, MD, USA).

### Immunoperoxidase‐based immunohistochemistry

After deparaffinization and rehydration, MSG tissue sections (3 μm thick) were boiled for 10 min. in sodium citrate buffer (10 mM, pH 6.0) for antigen retrieval and treated with 3% H_2_O_2_ in methanol for 15 min. at room temperature to block endogenous peroxidase activity. Sections were then washed and incubated with Ultra V block (UltraVision Large Volume Detection System Anti‐Polyvalent, HRP, catalogue number TP‐125‐HL; LabVision, Fremont, CA, USA) for 10 min. at room temperature according to the manufacturer's protocol. After blocking non‐specific site binding, slides were incubated overnight at 4°C with mouse monoclonal anti‐human podoplanin antibody (D2‐40) (1:50 dilution; catalogue number M3619; Dako) diluted in 1% BSA in PBS. The day after, tissue sections were washed three times in PBS and incubated with biotinylated secondary antibodies (UltraVision Large Volume Detection System Anti‐Polyvalent, HRP; LabVision) for 10 min. at room temperature. Subsequently, the slides were washed three times in PBS and incubated with streptavidin peroxidase (UltraVision Large Volume Detection System Anti‐Polyvalent, HRP; LabVision) for 10 min. at room temperature. Immunoreactivity was developed using 3‐amino‐9‐ethylcarbazole (AEC kit, catalogue number TA‐125‐SA; LabVision) as chromogen (brownish‐red colour). Minor salivary gland sections were finally counterstained with Mayer's hematoxylin (Bio‐Optica, Milan, Italy), washed, mounted in an aqueous mounting medium and observed under a Leica DM4000 B microscope equipped with fully automated transmitted light axes (Leica Microsystems). Sections not exposed to primary antibodies or incubated with isotype‐matched and concentration‐matched non‐immune mouse IgG (Sigma‐Aldrich) were included as negative controls for antibody specificity. Light microscopy images were captured with a Leica DFC310 FX 1.4‐megapixel digital colour camera equipped with the Leica software application suite LAS V3.8 (Leica Microsystems).

### Determination of numbers of lymphatic vessels

D2‐40‐immunopositive lymphatic vessels were counted in five randomly chosen microscopic high‐power fields (hpf; ×40 original magnification) of the interlobular MSG connective tissue per sample by two independent investigators blinded to MSG biopsy classification. The mean of the two different observations for each sample (8 normal, 8 NSCS and 12 pSS MSG samples) was used for analysis.

### Statistical analysis

SPSS V.21.0 package (Statistical Package for the Social Sciences, Chicago, IL, USA) was used, and either Mann–Whitney *U*‐test or Spearman's rho correlation coefficient was performed. All values are presented as mean ± S.E.M. Values of *P* < 0.05 were considered statistically significant.

## Results

### Circulating LEPCs are expanded in pSS and correlate with serum VEGF‐C, serum IL‐17 and circulating Th17 cell percentage

**Figure 1:**
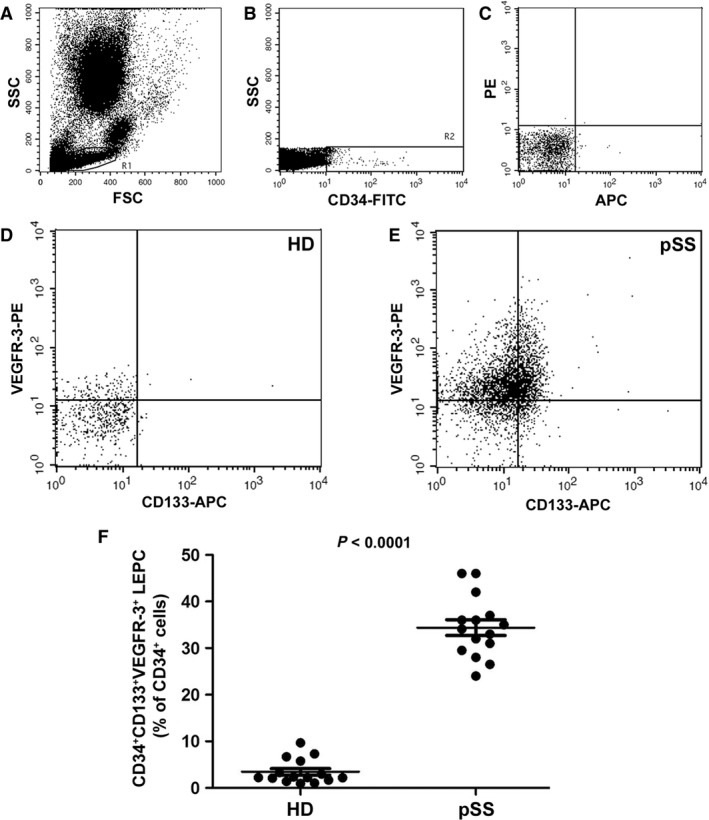
Evaluation of lymphatic endothelial precursor cells (LEPCs) in the peripheral blood of patients with primary Sjögren's syndrome (pSS) and healthy donors (HD). CD34^+^ CD133^+^ VEGFR‐3^+^ LEPCs were identified by flow cytometry within the lymphocyte gate **(A)**. **(B)** Representative flow cytometry dot plot displaying the gate drawn to select total CD34^+^ cells among which CD133^+^ VEGFR‐3^+^ cells (LEPCs) were identified. **(C)** Isotype controls. **(D** and **E)** Representative flow cytometry dot plots of one HD **(D)** and one patient with pSS **(E)**. **(F)** CD34^+^ CD133^+^ VEGFR‐3^+^ LEPCs (expressed as % of total CD34^+^ cells) are significantly increased in the peripheral blood of patients with pSS compared with HD. Data are shown as dot plots with mean ± S.E.M. Each dot represents a participant. Mann–Whitney *U*‐test was used for statistical analysis.

**Figure 2:**
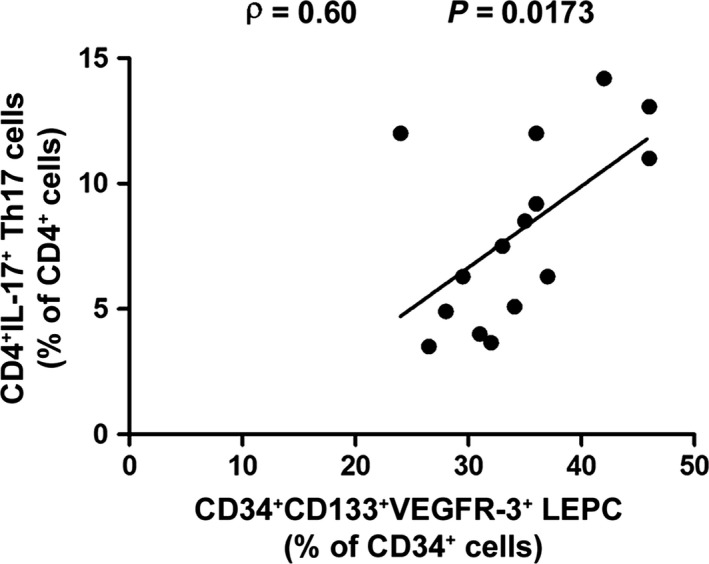
In the peripheral blood of patients with primary Sjögren's syndrome (pSS), the percentage of CD34^+^ CD133^+^ VEGFR‐3^+^ lymphatic endothelial precursor cells (LEPCs) is directly correlated to that of CD4^+^ IL‐17^+^ (Th17) cells as determined by Spearman's rho correlation coefficient. Data are shown as a scatterplot, each dot representing a patient.

**Figure 3:**
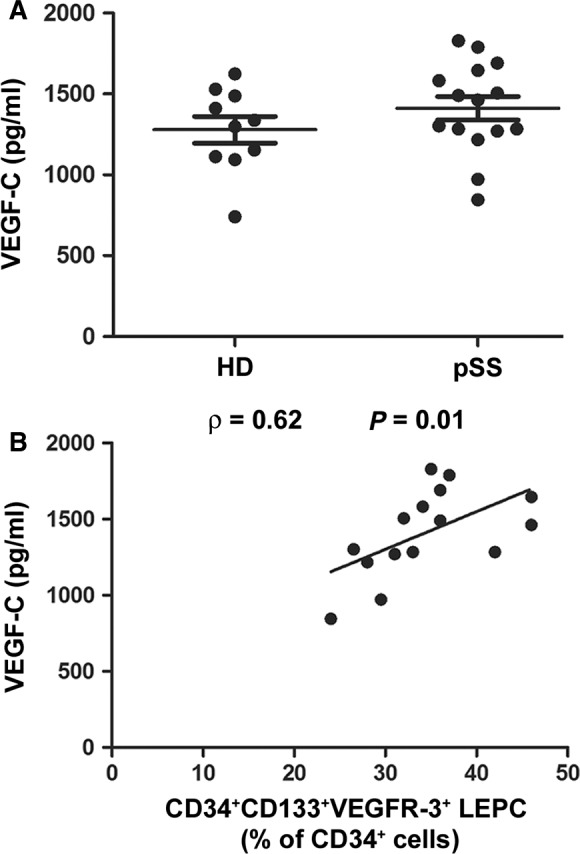
(**A**) Serum levels of VEGF‐C determined by ELISA. Serum VEGF‐C levels are comparable in patients with primary Sjögren's syndrome (pSS) and healthy donors (HD). Data are shown as dot plots with mean ± S.E.M. Each dot represents a participant. Mann–Whitney *U*‐test was used for statistical analysis. (**B**) In pSS, serum VEGF‐C concentration is directly correlated to the percentage of circulating CD34^+^ CD133^+^ VEGFR‐3^+^ lymphatic endothelial precursor cells (LEPCs) as determined by Spearman's rho correlation coefficient. Data are shown as a scatterplot, each dot representing a patient.

Demographic, clinical and serological features of the cohort of patients with pSS enrolled for analyses on peripheral blood samples are summarized in Table 1. We first sought to investigate the proportion of circulating LEPCs in pSS patients with respect to HD. Lymphatic endothelial precursor cells were identified within the lymphocyte gate (Fig. 1A) as CD34^+^CD133^+^VEGFR‐3^+^ cells (Fig. 1B–E). Patients with pSS displayed significantly higher proportions of circulating LEPCs compared to HD (*P* < 0.0001; Fig. 1F). A representative plot of one HD and one patient with pSS is displayed in Figure 1D and E, respectively. In addition, the percentage of circulating LEPCs was directly correlated to that of Th17 cells, identified as CD4^+^IL‐17^+^ lymphocytes (Spearman's rho = 0.60, *P* = 0.0173; Fig. 2). Subsequently, we assessed the concentration of VEGF‐C in paired serum samples and observed comparable levels of this molecule in pSS and HD (Fig. 3A). Of interest, however, VEGF‐C concentration was directly correlated to LEPC percentage in patients with pSS (Spearman's rho = 0.62, *P* = 0.01; Fig. 3B). Finally, the concentration of IL‐17 resulted directly correlated to the percentage of circulating LEPCs in paired serum samples from patients with pSS (Spearman's rho = 0.53, *P* = 0.04).

**Table 1:** Demographic, clinical and serological features of patients with primary Sjögren's syndrome (pSS) enrolled for analyses on peripheral blood samples

Number of patients	15
Age, years^a^	56 ± 3
Disease duration, years^a^	12 ± 2
Xerostomia	14 (93)
Xerophthalmia	15 (100)
Salivary gland enlargement	6 (40)
Extraglandular manifestations^b^	10 (67)
Lymphoma	0
Hypocomplementemia	10 (67)
Leukopenia	2 (13)
Hypergammaglobulinemia	9 (60)
Antinuclear antibodies	15 (100)
Autoantibodies	
Neither anti‐Ro/SSA nor anti‐La/SSB	3 (20)
Anti‐Ro/SSA only	6 (40)
Anti‐Ro/SSA and anti‐La/SSB	6 (40)
Rheumatoid factor	8 (53)
Hydroxychloroquine 200 mg/day	8 (53)
ESSDAI, median (range)	2 (0–4)

a These values are reported as mean ± S.E.M. Unless otherwise stated, all other values are reported as number (percentage) of patients.

b Eight patients with articular involvement and two patients with Raynaud's phenomenon. All other extraglandular manifestations were ruled out.

### Lymphatic vascularization and expression of lymphangiogenic mediators are increased in MSGs from patients with pSS

**Figure 4:**
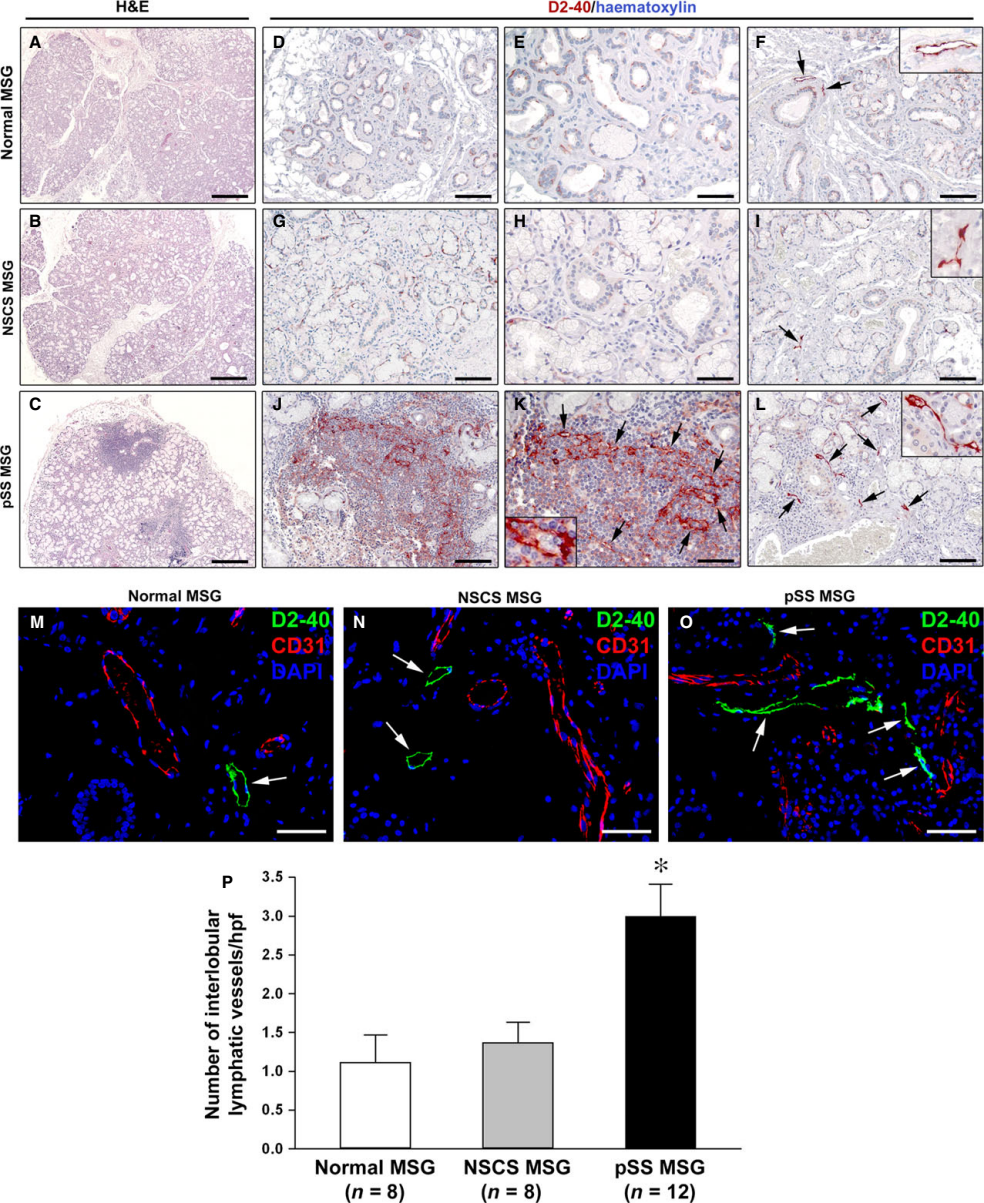
Histopathology and characterization of lymphatic vascularization of minor salivary glands (MSGs). (**A**,** D**,** E** and **F**) Normal MSGs. (**B**,** G**,** H** and **I**) MSGs from non‐specific chronic sialadenitis (NSCS). (**C**,** J**,** K** and **L**) MSGs from primary Sjögren's syndrome (pSS). (**A**–**C**) Hematoxylin and eosin staining. pSS MSGs display periductal inflammatory aggregates (foci) replacing the secretory units. (**D**–**L**) Immunoperoxidase staining for the lymphatic vessel marker podoplanin (D2‐40; brownish‐red) with hematoxylin counterstain. In normal and NSCS MSGs, lymphatic vessels are absent from acinar regions (**D**,** E**,** G** and **H**), while a few lymphatic vessels (*arrows*) are present in the interlobular connective tissue, especially around excretory ducts (**F** and **I**). The *insets* show higher magnification views of lymphatic vessels from the corresponding panels. In pSS MSGs, a newly formed lymphatic capillary network (*arrows*) is present within periductal inflammatory infiltrates (**J** and **K**). One lymphatic capillary from the corresponding panel is shown at higher magnification in the *inset*; note the presence of some lymphocytes within the lumen. Numerous lymphatic vessels (*arrows*) are observed in the interlobular connective tissue of pSS MSGs (**L**). The *inset* shows a higher magnification view of a periductal lymphatic vessel from the corresponding panel. (**M**–**O**) Double immunofluorescence staining for D2‐40 (green) and CD31 (red) with 4′,6‐diamidino‐2‐phenylindole (DAPI, blue) counterstain for nuclei. MSG lymphatic vessels are strongly immunostained by D2‐40 (*arrows*), while CD31^+^ blood vessels are negative for D2‐40. Original magnification: ×5 (**A**–**C**), ×20 (**D**,** F**,** G**,** I**,** J** and **L**), ×40 (**E**,** H**,** K** and **M**–**O**), ×63 (**F**,** I**,** K** and **L** insets). Scale bar: 400 μm (**A**–**C**), 100 μm (**D**,** F**,** G**,** I**,** J** and **L**), 50 μm (**E**,** H**,** K** and **M**–**O**). (**P**) Quantification of interlobular lymphatic vessels in normal (*n* = 8), NSCS (*n* = 8) and pSS (*n* = 12) MSGs. Data are mean ± S.E.M. of D2‐40‐positive lymphatic vessel counts per high‐power field (hpf). *P < 0.01 *versus* normal and NSCS MSGs (Mann–Whitney *U*‐test).

**Figure 5:**
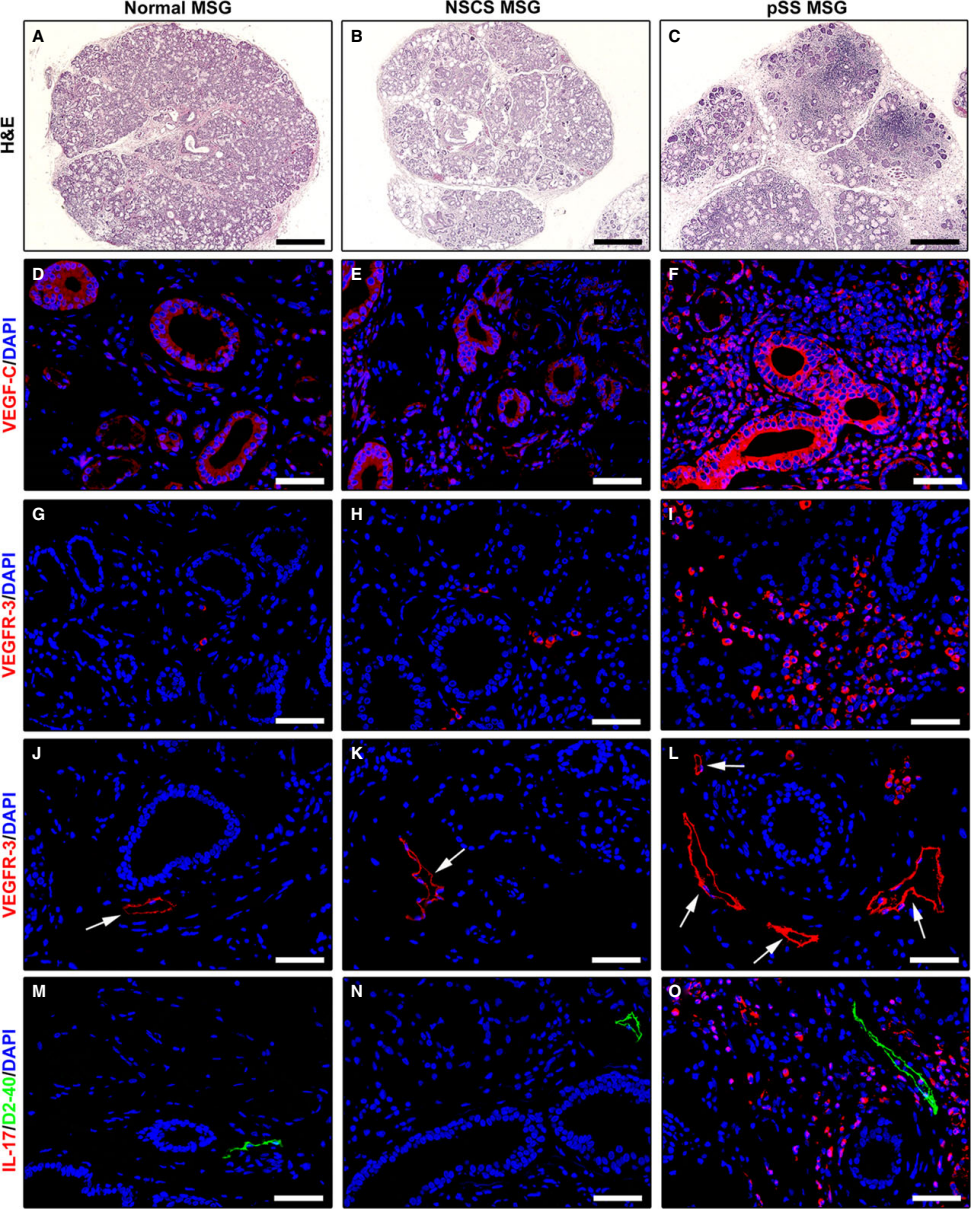
Increased expression of lymphangiogenic mediators in minor salivary glands (MSGs) from patients with primary Sjögren's syndrome (pSS). (**A, D, G, J** and **M**) Normal MSGs. (**B, E, H, K** and **N**) MSGs from non‐specific chronic sialadenitis (NSCS). (**C, F, I, L** and **O**) MSGs from pSS. (**A–C**) Hematoxylin and eosin staining. pSS MSGs display periductal inflammatory aggregates (foci) replacing the secretory units. (**D–F**) Immunofluorescence staining for VEGF‐C (red) with 4′,6‐diamidino‐2‐phenylindole (DAPI, blue) counterstain for nuclei. Faint expression of VEGF‐C is detected in normal and NSCS MSGs (**D** and **E**). In pSS MSGs, VEGF‐C is strongly expressed in ductal epithelial cells, microvessels and periductal inflammatory cells (**F**). (**G–L**) Immunofluorescence staining for VEGF receptor (VEGFR)‐3 (red) with DAPI (blue) counterstain. Numerous VEGFR‐3^+^ infiltrating mononuclear cells are present in pSS MSGs (**I**). Both in normal and NSCS MSGs, lymphatic capillaries (*arrows*) show weak VEGFR‐3 positivity (**J** and **K**). VEGFR‐3 expression is strongly increased in lymphatic capillaries (*arrows*) of pSS MSGs (**L**). (**M–O**) Double immunofluorescence staining for interleukin (IL)‐17 (red) and the lymphatic vessel marker podoplanin (D2‐40, green) with DAPI (blue) counterstain. No IL‐17 expression can be detected either in normal or NSCS MSGs (**M** and **N**). Numerous IL‐17^+^ inflammatory cells are present around lymphatic vessels in pSS MSGs (**O**). Original magnification: ×5 (**A–C**), ×40 (**D–O**). Scale bar: 400 μm (**A–C**), 50 μm (**D–O**).

Immunohistological analyses were carried out on labial MSG biopsies from 12 patients with pSS (*i.e*. displaying FLS) and 16 sicca syndrome non‐pSS controls. Of these, eight participants displayed normal MSGs and eight displayed a certain degree of MSG inflammation (*i.e*. NSCS), but no evidence of FLS. Representative images of hematoxylin‐ and eosin‐stained MSG sections are shown in Figures 4A–C and 5A–C.

For specific detection of lymphatic vessels, we performed both immunoperoxidase‐based immunohistochemistry (Fig. 4D–L) and immunofluorescence (Fig. 4M–O) using the mouse monoclonal antibody D2‐40 that reacts with a fixation‐resistant epitope in podoplanin, a mucin‐type transmembrane protein expressed at high levels in lymphatic ECs but not in blood vascular ECs 22. As displayed in Figure 4M–O, MSG lymphatic vessels were consistently and strongly immunostained by D2‐40, while CD31^+^ blood vessels were consistently negative for D2‐40. In line with previous observations in human parotid gland and MSGs 7, lymphatic vessels were absent from acinar regions in control MSGs (Fig. 4D, E, G and H). Indeed, lymphatic vessels were only detected in the interlobular connective tissue, especially around excretory ducts, in both normal and NSCS MSGs (Fig. 4F and I). In pSS MSGs, a newly formed lymphatic capillary network was found within periductal inflammatory infiltrates replacing the secretory units (Fig. 4J and K). Moreover, the lymphatic vessel network was expanded in the interlobular connective tissue of pSS MSGs (Fig. 4L). In fact, the number of interlobular lymphatic vessels was significantly increased in pSS MSGs compared with normal and NSCS MSGs (both *P* < 0.01; Fig. 4M–P).

**Figure 6:**
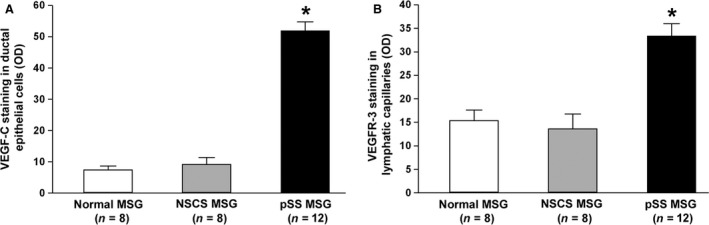
(**A**) Densitometric analysis of VEGF‐C immunofluorescent staining in ductal epithelial cells of primary Sjögren's syndrome (pSS), normal and non‐specific chronic sialadenitis (NSCS) minor salivary glands (MSGs). (**B**) Densitometric analysis of VEGF receptor (VEGFR)‐3 immunofluorescent staining in lymphatic capillaries of primary pSS, normal and NSCS MSGs. Data are mean ± S.E.M. of optical density (OD) in arbitrary units and were obtained from 8 normal, 8 NSCS and 12 pSS MSG specimens. *P < 0.01 *versus* normal and NSCS MSGs (Mann–Whitney *U*‐test).

As far as lymphangiogenic mediators are concerned, VEGF‐C was barely detectable in ductal epithelial cells and microvessels of both normal and NSCS MSGs (Fig. 5D and E). Conversely, in pSS MSGs, a strong expression of VEGF‐C was detected in ductal epithelial cells, microvessels and periductal inflammatory cells (Fig. 5F). As displayed in Figure 6A, densitometric analysis showed that VEGF‐C immunofluorescent staining intensity was significantly increased in ductal epithelial cells of pSS MSGs compared with normal and NSCS MSGs (both *P* < 0.01). Numerous VEGFR‐3^+^ infiltrating mononuclear cells were exclusively observed in pSS MSGs (Fig. 5G–I). In addition, the expression of VEGFR‐3 was strongly increased in lymphatic capillaries of pSS MSGs with respect to normal and NSCS MSGs (Fig. 5J–L). Indeed, VEGFR‐3 immunofluorescent staining intensity was significantly greater in pSS MSG lymphatic ECs than in normal and NSCS MSG lymphatic ECs (both *P* < 0.01; Fig. 6B). Finally, IL‐17^+^ inflammatory cells were preferentially observed around lymphatic vessels in pSS MSGs, while no IL‐17 expression could be detected either in normal or NSCS MSGs (Fig. 5M–O).

## Discussion

Since lymphatic neovascularization is a key feature of chronic inflammation, a role of this process in inflammatory autoimmune diseases has been speculated 3. In this study, we investigated for the first time lymphvasculogenesis and lymphangiogenic mediators, namely LEPCs and VEGF‐C/VEGFR‐3 axis 9, 10, 11, 23, in pSS and demonstrated that they are increased in this disease. In addition, we reported an increased and anatomically aberrant lymphatic neovascularization in MSGs from patients with pSS. The only two available studies that focused on lymphatic neovascularization in MSGs were limited to the assessment of glandular mature lymphatic ECs and yielded conflicting results 7, 8. In fact, while Yazisiz *et al*. reported an increase of lymphatic capillaries within the mononuclear cell infiltrate of MSGs from eight patients with pSS 8, McCall and Baker failed to identify any differences between patients and controls 7. As far as MSGs are concerned, our present study not only assessed lymphatic vessel distribution but also analysed for the first time the expression of the pro‐lymphangiogenic VEGF‐C/VEGFR‐3 axis. Interestingly, the increased expression of VEGFR‐3 consistently observed in lymphatic endothelium further indicates an activation of the pro‐lymphangiogenic programme within the inflammatory milieu of pSS MSGs.

Besides being involved in tissue fluid homeostasis and lipid uptake in the gastrointestinal tract, lymphatic vessels also play a role in immune surveillance driving the recirculation of immune cells toward secondary lymphoid organs. In normal conditions, salivary gland lymphatic vessels are selectively localized within the interlobular connective tissue, being absent in lobules and acinar regions 7, 24. Our study revealed that pSS salivary gland tissues display a consistent increase of interlobular lymphatic vessels. In addition, it was of great interest the finding that lymphatic vessels were also evident where the mononuclear cell infiltrates replaced acinar structures. Such *de novo* lymphatic network may account for a recirculation of immune cells from MSGs and might even help autoreactive lymphocytes in reaching extraglandular sites. Moreover, the strong glandular expression of VEGF‐C by ductal epithelial cells may be an additional clue for the active role of glandular epithelium in the scenario of disease pathogenesis, possibly by recruiting LEPCs from bone marrow to generate new glandular lymphatic vessels *via* lymphvasculogenesis. In addition, we observed strong VEGF‐C expression in pSS periductal inflammatory cells, presumably monocytes/macrophages. Interestingly, monocytes/macrophages have been proposed to participate in lymphangiogenesis in at least two ways, namely as a source of VEGF‐C after appropriate stimulation or by transdifferentiation into lymphatic ECs that integrate into the growing capillaries 25. Indeed, as potential lymphangioblastic precursors, a large pool of CD14^+^ monocytes was identified in the circulation. These monocytes were found to constitutively express VEGFR‐3 and turn on expression of podoplanin and several other lymphatic markers when cultured for a prolonged period 25. In this context, it is noteworthy the presence of numerous VEGFR‐3^+^ infiltrating mononuclear cells found in pSS MSGs.

Of note, our study also sheds light on a possible association between lymphvasculogenic/lymphangiogenic mediators and IL‐17 axis in pSS. Interleukin‐17 is a pro‐inflammatory cytokine representing a leading player in the pathogenesis of systemic autoimmune diseases, including pSS 13, 26. Interleukin‐17 signalling in immune and non‐immune target cells induces a cascade of events that culminates in the release of pro‐inflammatory mediators. In the matter of lymphatic neovascularization, a previously published study provided evidence that IL‐17 also displays a pro‐lymphangiogenic function by stimulating LEPCs and fostering their differentiation into mature lymphatic ECs 17. In pSS, IL‐17 is able to trigger FLS 13 and is associated with the extent of glandular inflammation 16, 27 and, possibly, with ectopic lymphoid neogenesis 16. Therefore, such a cytokine appears to represent an intriguing therapeutic target in this disease. Herein, we demonstrated that circulating IL‐17 and Th17 cells are directly correlated to LEPCs, thereby suggesting a possible pro‐lymphangiogenic/lymphvasculogenic activity of this cytokine in pSS. Taken this association and the role of IL‐17 in pSS, it is reasonable to postulate that aberrant glandular lymphatic neovascularization in pSS is another facet of disease pathogenesis.

In conclusion, our study demonstrates for the first time that lymphvasculogenesis and lymphangiogenesis are active in pSS and suggests another pathogenic role of IL‐17 in this disease. We are aware that functional *in vitro* studies will be required to confirm that IL‐17 may directly affect LEPC activation and differentiation in pSS. However, we believe that the present data unmasked a novel aspect of disease pathogenesis, providing the basis for further investigation on this issue in larger patient cohorts and identifying another possible rationale for therapeutic targeting of IL‐17 in pSS 28.

## Conflicts of interest

The authors confirm that there are no conflicts of interest.

## Author contribution

Design of the study: AA and MM; acquisition of data: AA, LI‐M, OB, IR, SC, RG and MM; interpretation of data: AA, LI‐M, RG and MM; and manuscript preparation: AA, LI‐M, RG and MM.
